# Downregulation of LIM kinase 1 suppresses ocular inflammation and fibrosis

**Published:** 2008-10-30

**Authors:** Matvey Gorovoy, Takahisa Koga, Xiang Shen, Zhengping Jia, Beatrice Y. Yue, Tatyana Voyno-Yasenetskaya

**Affiliations:** 1 Department of Pharmacology, College of Medicine, University of Illinois at Chicago, Chicago, IL; 2 Department of Ophthalmology and Visual Sciences, College of Medicine, University of Illinois at Chicago, Chicago, IL; 3 Neuroscience and Mental Health Program, Hospital for Sick Children, Toronto, Ontario, Canada

## Abstract

**Purpose:**

The purpose of this study was to determine if downregulation of LIM kinase 1 (*LIMK1*) by genetic deletion or direct application of *LIMK1*-targeted siRNA could suppress TGF-β mediated ocular inflammation and fibrosis.

**Methods:**

*LIMK1* specific siRNAs designed from the human sequence were transfected into human corneal fibroblasts in culture. Immunofluorescence and immunoblotting were performed to examine the fibronectin assembly. The effects of *LIMK1* downregulation on actin cytoskeleton organization and focal adhesion formation were studied. A wound closure assay was used to assess cell migration in in vitro fibroblast cultures. The in vivo effects of *LIMK1* genetic deletion or downregulation by mouse siRNA were evaluated in a mouse model of ocular inflammation generated by subconjunctival injection of phosphate buffered saline and latex beads. Cellularity on tissue sections was examined after staining with hematoxylin and eosin. Anti-CD45 antibody was used for the leukocyte detection.

**Results:**

Downregulation of *LIMK1* in cultured corneal fibroblasts impaired fibronectin secretion and assembly, diminished actin polymerization and focal adhesion formation, and retarded cell migration. In the mouse model of ocular inflammation, both genetic deletion and downregulation of *LIMK1* by siRNA significantly reduced inflammatory response.

**Conclusions:**

Downregulation of *LIMK1* was efficacious to decrease the ocular inflammation. We disclose a possibility that *LIMK1* may mediate TGF-β-dependent signaling during ocular inflammation. A direct application of siRNA into eyes to downregulate *LIMK1* expression may provide a novel therapy for suppression and prevention of ocular inflammation and fibrosis.

## Introduction

LIM kinase 1 (LIMK1) is a serine/threonine kinase that regulates microtubule stability and actin polymerization [1]. LIMK1 promotes actin polymerization by phosphorylation and inactivation of the actin depolymerization factor – cofilin [2,3]. It also negatively regulates microtubule dynamics and assembly via phosphorylation of p25/TPPP [4]. LIMK1 is activated via phosphorylation by downstream effectors of small GTPases: Rho kinase (ROCK) [5]; p21 protein (Cdc42/Rac)-activated kinase (PAK1) [6]; and PAK4 [7].

Transforming growth factor-β (TGF-β), a family of cytokines, is known to be a key mediator of fibrotic responses such as fibronectin deposition and cell migration to wounding site [8]. This factor has been implicated in a variety of conditions that include proliferative vitreoretinopathy [9], cataract formation [10], corneal opacities [11], and subconjunctival scarring, a complication of filtration surgery in glaucoma [12,13].

There have been reports of cross-talk between LIMK1 and TGF-β receptor superfamilies. A direct association between LIMK1 and bone morphogenetic protein receptor type II (BMPR-II), a member of the TGF-β superfamily, mediated actin cytoskeleton dynamics [14,15]. It has also been shown that TGF-β type I receptor can indirectly activate LIMK2, a member of the LIMK family, through Rho and its downstream effector ROCK1 [16] to regulate actin assembly.

In the glaucoma filtration surgery, postoperative fibrosis or scarring at the wound site is a critical determinant of the surgical outcome [17,18]. Although anti-scarring agents such as mitomycin C and 5-fluorouracil can prevent post-operative scarring and improve surgical outcome [19,20], they cause widespread fibroblast cell death and are often associated with severe and potentially blinding complications [21,22]. Therefore targeting one of the pro-inflammatory pathways via siRNA-dependent protein downregulation might be an effective strategy to reduce ocular inflammation and fibrosis.

We have recently determined that LIMK1 plays a pro-inflammatory role in mouse lungs via disruption of endothelial barrier function and promotion of leukocyte diapedesis through regulation of cytoskeleton dynamics (unpublished data). The important role of LIMK1 during inflammatory response and its possible cross-talk with TGF-β have led us to hypothesize that LIMK1 may be involved in inflammation through TGF-β signaling, and that downregulation of *LIMK1* might be an effective strategy to suppress ocular inflammation and fibrosis.

In the current study, the RNA interference and genetic deletion approaches were employed to test our hypothesis. We showed here that downregulation of *LIMK1* in human corneal fibroblasts led to a significant decrease in fibronectin deposition. The actin stress fibers and focal adhesions were diminished and the fibroblast migration was retarded. Moreover, downregulation of *LIMK1* in a mouse model via both genetic deletion and direct application of *LIMK1*-targeted siRNA in the eyes markedly reduced ocular inflammation.

## Methods

### Cell cultures

Normal human corneas from donors aged 13, 29, 34, 45, and 47 years were obtained from the Illinois Eye Bank (Chicago, IL). The procurement of tissues was approved by the Institutional Review Board at the University of Illinois at Chicago in compliance with the declaration of Helsinki. The endothelium-Descemet’s membrane was stripped off under a dissecting microscope. The stroma was then mechanically separated from the epithelium-stroma and used as an explant to initiate corneal fibroblast cultures. The cells were maintained in Dulbecco's modified Eagle's minimum essential medium (MEM) supplemented with glutamine, 10% fetal calf serum, 5% calf serum, nonessential and essential amino acids, and antibiotics as previously described [23]. All of the in vitro experiments were repeated at least 3 times. Results were confirmed with second- or third-passaged cells derived independently from at least 3 different donors.

### *LIMK1* siRNA sequences

Double-stranded siRNA targeted against human *LIMK1*: CCU GGA GGG AAG AAC GUA UUU, and mismatch siRNA CCU GAA AGA AAA AAC GUA UUU (where 4 nucleotides were mutated G/A) were from Dharmacon (Chicago, IL). The siRNA was described previously [1]. The specificity of the *LIMK1* siRNA was verified or the siRNA study was validated by using 1) mismatch controls, where mutation of only several nucleotides completely abolished the silencing effect; and 2) several siRNAs targeted against different regions on *LIMK1* mRNA, which showed similar silencing effects. The double-stranded mouse counterpart of the *LIMK1* siRNA: ACC GGA UCU UGG AAA UCA AUU, and negative control siRNA were from Qiagen (Valencia, CA).

### Transfection of siRNA duplexes

Normal human corneal fibroblasts were transfected with *LIMK1* specific siRNA duplex (50 or 100 nM final concentration) or scrambled siRNA using TransIT-TKO reagent (Takara Mirus, Madison, WI) according to the manufacturer’s protocol. Scrambled siRNA was used as a negative control at 100 nM concentration in all experiments. The cells were harvested 48 or 72 h after transfection for analysis. Also as controls, corneal fibroblasts were either untreated or treated only with TransIT-TKO.

### Fibronectin, actin, and paxillin staining

Corneal fibroblasts were plated at a confluent or sub-confluent density onto 8 well glass chamber slides (Nalge Nunc International, Naperville, IL) without extracellular matrix (ECM) coating. The cells were transfected the next day and were fixed in cold methanol without permeabilization to examine the fibronectin network 48 or 72 h after transfection. For actin and paxillin staining, cells were fixed in paraformaldehyde-lysine-phosphate buffer and permeabilized in 0.2% Triton X-100. To carry out immunostaining, corneal fibroblasts were incubated with polyclonal rabbit anti-human fibronectin (1:100; BD Biosciences, Lexington, KY) or monoclonal anti-paxillin (1:100; Millipore, Billercia, MA). FITC-goat anti-rabbit (1:200) or Cy3-goat anti-mouse (1:200; Jackson ImmunoResearch, West Grove, PA) IgG was used as the secondary antibody. The actin structure was visualized after incubation for 40 min with Alexa 488-phalloidin (1:40; Molecular Probes, Eugene, OR). The nuclei of the cells were counterstained with 4’,6’-diamidino-2-phenylindole dihydrochloride (DAPI). The slides were examined under a Zeiss 100M microscope.

### Western blotting

After siRNA transfection, corneal fibroblasts were lysed in a Triton lysis buffer (20 mM Tris-HCl, pH 7.5, 100 mM NaCl, 1 mM EDTA, and 1% v/v Triton X-100). Proteins in cell lysates were quantified by bicinchoninic acid (BCA) protein assay (Pierce, Rockford, IL) with BSA as a standard. Equal amounts of proteins (20 µg/lane) were resolved on 10% sodium dodecyl sulfate (SDS)-polyacrylamide gels. The proteins were then transferred to nitrocellulose membranes for probing with rabbit anti-LIMK1 antibody (1:500; Cell Signaling Technology Inc., Danvers, MA) and horseradish peroxidase (HRP) conjugated goat anti-rabbit IgG (1:5000; Jackson ImmunoReseach). As a protein loading control, the membranes were also probed with anti-glyceraldehyde 3-phosphate dehydrogenase (anti-GAPDH; Trevigen, Gaithersburg, MD). Signals were detected by chemiluminescence. The gel image captured with Gel Doc 2000 image analyzer (Bio-Rad, Hercules, CA) was analyzed by densitometry using Image station 440 (Eastman Kodak Company, Rochester, NY). Band intensities were determined and the ratios of LIMK1 intensity to that of GAPDH were calculated.

For secreted fibronectin, 72 h following transfection, cells were incubated with serum-free media for 24 h. After normalizing against the protein content in cell lysates, equal aliquots of media samples were electrophoresed on 10% SDS polyacrylamide gels under reducing conditions. Immunoblotting was performed using rabbit anti-human fibronectin (1:5000) and HRP-goat anti-rabbit IgG (1:10000; Jackson Immunoresearch).

### Wound closure assay for cell migration

Seventy-two hours after transfection, corneal fibroblasts were scratched with a sterile P20 pipette tip [24]. The migration of cells into the wound was examined by phase-contrast microscopy 7 h after wounding. Total area of the wound in each 10X field and the area covered by the migrating cells within the wound were measured using the Image Processing Tool Kit (Version 3.0; Reindeer Graphics, Ashville, NC). At least 10 fields were analyzed and the mean percentage of wound area was calculated. Student’s *t*-test was used for the statistical analysis.

### Mouse model of subconjunctival inflammation

*LIMK1* deficient mice were described elsewhere [25]. The experimental protocol was approved by the animal care committee at the University of Illinois at Chicago. Animal care guidelines comparable to those published by the US Public Health Service were followed. Mice underwent general anesthesia with intraperitoneal injections of ketamine and xylazine. The subconjunctival scarring model was generated similar to that reported previously [26], with modifications [8]. In brief, 10 µl of phosphate buffered saline (PBS) containing latex beads (1.053 µm diameter, 300 µg/ml; Polysciences, Warrington, PA) was injected to the temporal subconjunctival space of mouse eyes. For experiments with siRNA, the left eyes of mice were injected in a masked manner with 10 µl of PBS/beads mixed with 0.1 µl of TransIT-TKO and 100 nM of *LIMK1* siRNA or scrambled siRNA. To serve as controls, the left eyes of other mice were either untreated, or injected with PBS/latex beads or with TransIT-TKO reagent alone. All the right eyes were untouched. Mice were sacrificed by cervical dislocation 2 days after injection. At least 5mice were used for each experiment.

The enucleated eyes were fixed with 10% PBS-buffered formalin and 5 µm thick paraffin sections were prepared. The sections were stained with hematoxylin and eosin to assess the inflammatory reaction [26]. The number of round and large inflammatory cells (as opposed to the long and thin resident fibroblasts) in subconjunctival areas of the sections was counted. The value was normalized to number of inflammatory cells per 10,000 µm^2^ area underneath the conjunctival epithelium. Tissue sections were also deparaffinized and stained with anti-CD45 antibody (1:200; Santa Cruz Biotechnology, Santa Cruz, CA) and FITC-labeled goat anti-rabbit IgG (Jackson Immunoresearch) to detect leukocytes. The number of anti-CD45-positive cells in subconjunctival areas was determined as above.

## Results

### *LIMK1* siRNA inhibits fibronectin assembly and fibronectin secretion

**Figure 1:**
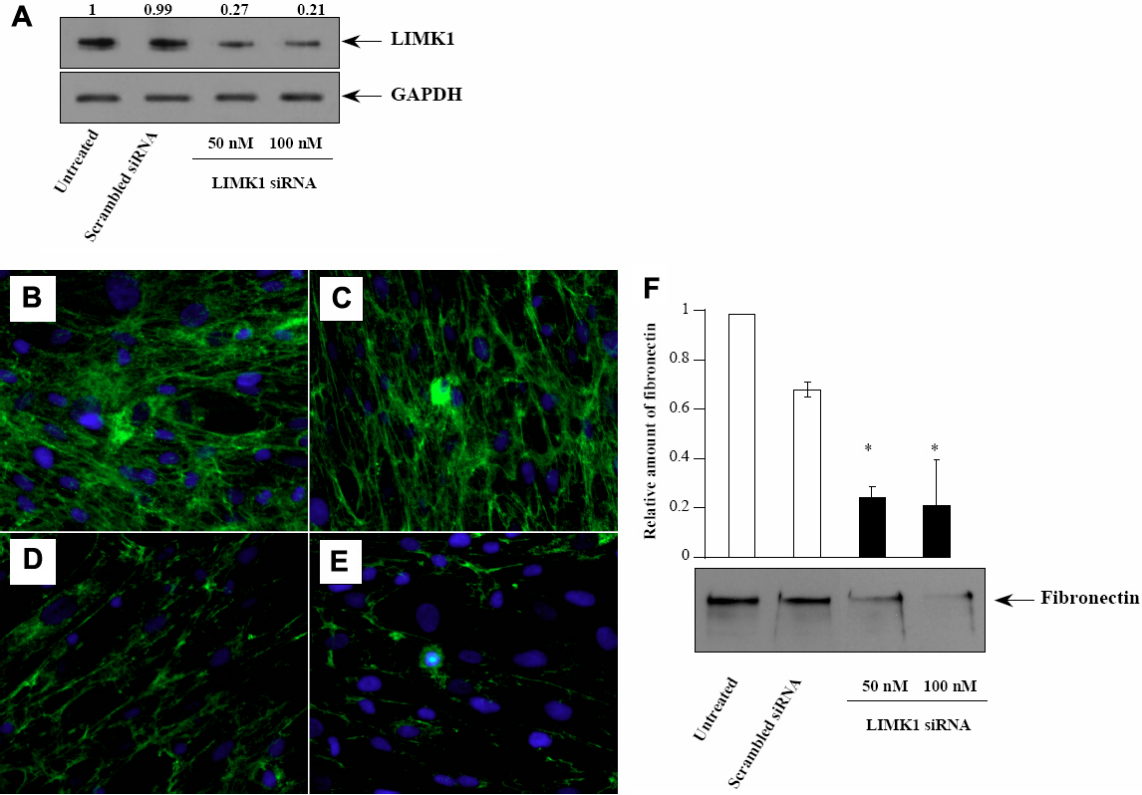
*LIMK1* siRNA downregulates LIMK1 protein and suppresses fibronectin deposition in human corneal fibroblasts. **A**: western blot analysis. Corneal fibroblasts were transfected as indicated for 72 h. Cells were lysed and protein levels were analyzed using LIMK1 and GAPGH antibodies. **B-E**: Immunofluorescence microscopy using fibronectin antibody and DAPI staining in fibroblast cultures (**B**) untreated; (**C**) treated with 100 nM scrambled control siRNA; (**D**) treated with 50 nM of *LIMK1*-targeted siRNA; or (**E**) treated with 100 nM of *LIMK1*-targeted siRNA. **F**: Quantitative analysis of fibronectin secreted to the culture media by western blotting. Samples were untreated or treated with control or *LIMK1*-targeted siRNA as indicated. Experiments were performed 3 times yielding similar results. Error bars represent standard deviation. The asterisk indicates a p<0.025 (n=3) compared to scrambled siRNA.

Endogenous LIMK1 was downregulated in human corneal fibroblasts using a *LIMK1*-specific siRNA at both 48 and 72 h post-transfection time points. This siRNA was used previously, proven to be effective in the 50–100 nM range in silencing *LIMK1* in human umbilical vein endothelial cells [1]. The siRNA knockdown of the endogenous LIMK1 in human corneal fibroblasts resulted in ~75% downregulation with 50 nM, and ~80% downregulation with 100 nM siRNA (Figure 1A).

Immunofluorescence microscopy was used to visualize fibronectin deposition. Untreated human corneal fibroblasts (Figure 1B) and those transfected with scrambled siRNA (Figure 1C) exhibited abundant fibronectin deposition and a substantial fibrillar network over cells (Figure 1B,C). The fibronectin deposition was significantly diminished in cultures 48 (data not shown) and 72 h (Figure 1D,E) after transfection with both 50 nM and 100 nM *LIMK1* siRNA. Cell density was similar in all samples, suggesting that the decreased fibronectin assembly was not related to the reduction in cell numbers.

To determine whether the effect of *LIMK1* siRNA on fibronectin fibrillogenesis was due to a decreased fibronectin secretion, we analyzed the amount of secreted fibronectin by western blotting (Figure 1F). Seventy-two hours after transfection with *LIMK1* siRNA, corneal fibroblasts, were incubated in serum-free medium for 24 h. The fibronectin in the media was detected as a 220 kDa protein band. Consistent with the immunofluorescence data, treatment with 50 nM or 100 nM *LIMK1* siRNA resulted in decreased fibronectin secretion into the culture; the reduction, up to 80%, was dose- dependent.

### *LIMK1* siRNA impairs corneal fibroblast migration and reduces actin polymerization and focal adhesion formation

**Figure 2:**
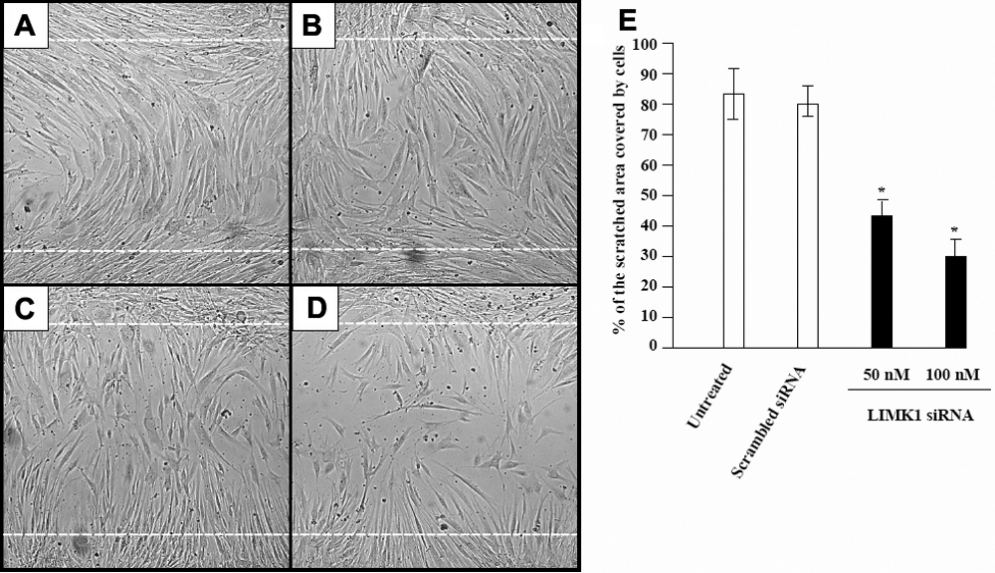
*LIMK1*-targeted siRNA blocks corneal fibroblast migration. **A-D**: Phase contrast micrographs demonstrating migration of (**A**) untreated cells; (**B**) cells treated with 100 nM scrambled control siRNA; (**C**) cells treated with 50 nM *LIMK1*-targeted siRNA; (**D**) cells treated with 100 nM *LIMK1*-tareted siRNA into the scratched wound area (marked by dotted white lines). **E**: Bar graph showing mean percentage of scratch-wounded area covered by migrating corneal fibroblasts in each specimen (n=10); error bars represent standard error of the mean. Asterisks denote values significantly different from those of samples treated with scrambled control siRNA (p<0.0001 [n=10] compared to scrambled siRNA). Experiments were repeated 3 times, yielding similar results.

A scratch wound closure assay was used to test whether downregulation of *LIMK1* would affect corneal fibroblasts motility. The data showed that corneal fibroblasts migrated into the wounded area (Figure 2A,B). Within 7 h, untreated (Figure 2A) and scrambled siRNA (100 nM)-transfected (Figure 2B) cells filled approximately 83% of the wound area (Figure 2E). Meanwhile, migration was significantly decreased in *LIMK1* siRNA treated cells. The percent of wound area covered was approximately 45% in the case of 50 nM (Figure 2C,E) and 28% in the case of 100 nM (Figure 2D,E) *LIMK1* siRNA.

**Figure 3:**
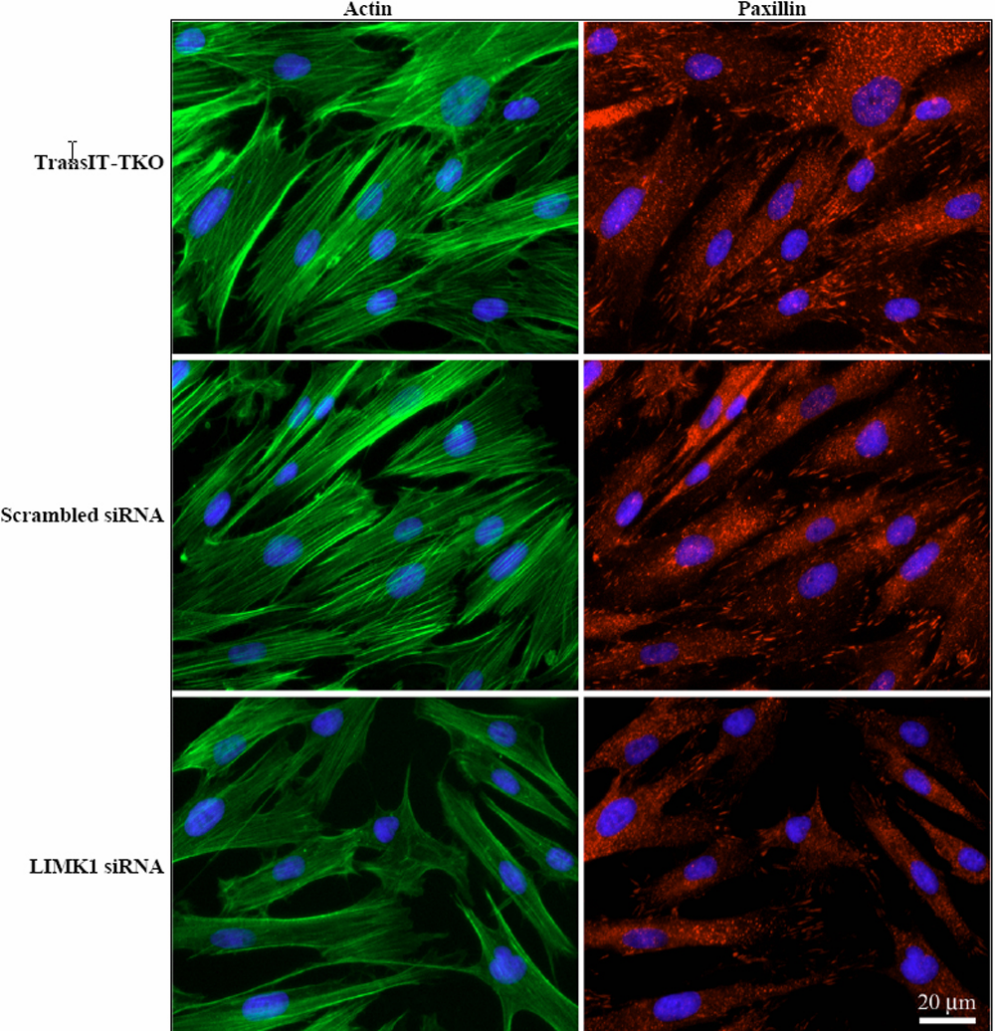
Downregulation of *LIMK1* decreases actin polymerization and focal adhesion formation. Corneal fibroblasts transfected with TransIT-TKO transfection reagent, a 100 nM scrambled or *LIMK1* siRNA for 72 h were fixed and permeablized. The cells were stained for actin (green color, using Alexa 488 phalloidin) and paxillin (red color, using anti-paxillin and Cy3-goat anti-mouse IgG) where indicated. The nuclei were stained with DAPI (blue color). Note the reduction of actin stress fibers and paxillin-positive focal adhesions in *LIMK1* siRNA-transfected cells. Bar, 20 μm.

As actin cytoskeleton dynamics and cell adhesion to the ECM are crucially involved in cell migration [27], we investigated whether actin dynamics and focal adhesion formation were affected upon downregulation of *LIMK1*. We observed that cells treated with transfection reagent TransIt-TKO alone as well as those transfected with scrambled siRNA all displayed prominent actin stress fibers. Treatment of *LIMK1* siRNA by contrast resulted in a substantial reduction of actin stress fibers (Figure 3). Staining for paxillin, a component of focal adhesions, was also decreased by *LIMK1* siRNA in cultured corneal fibroblasts (Figure 3).

### Downregulation of *LIMK1* via genetic deletion or siRNA reduces ocular inflammation in a mouse model

To generate a mouse model of inflammation and fibrosis, PBS and latex beads were injected into the subconjunctival space of mice [8]. This model is a modification of the previously described one [26], in which PBS only was used for injections. In our PBS/beads model, the inflammation response was significantly augmented as compared to PBS only [8]. The augmentation of inflammatory response was detected by the increased number of inflammatory cells per area underneath the conjunctival epithelium [8].

**Figure 4:**
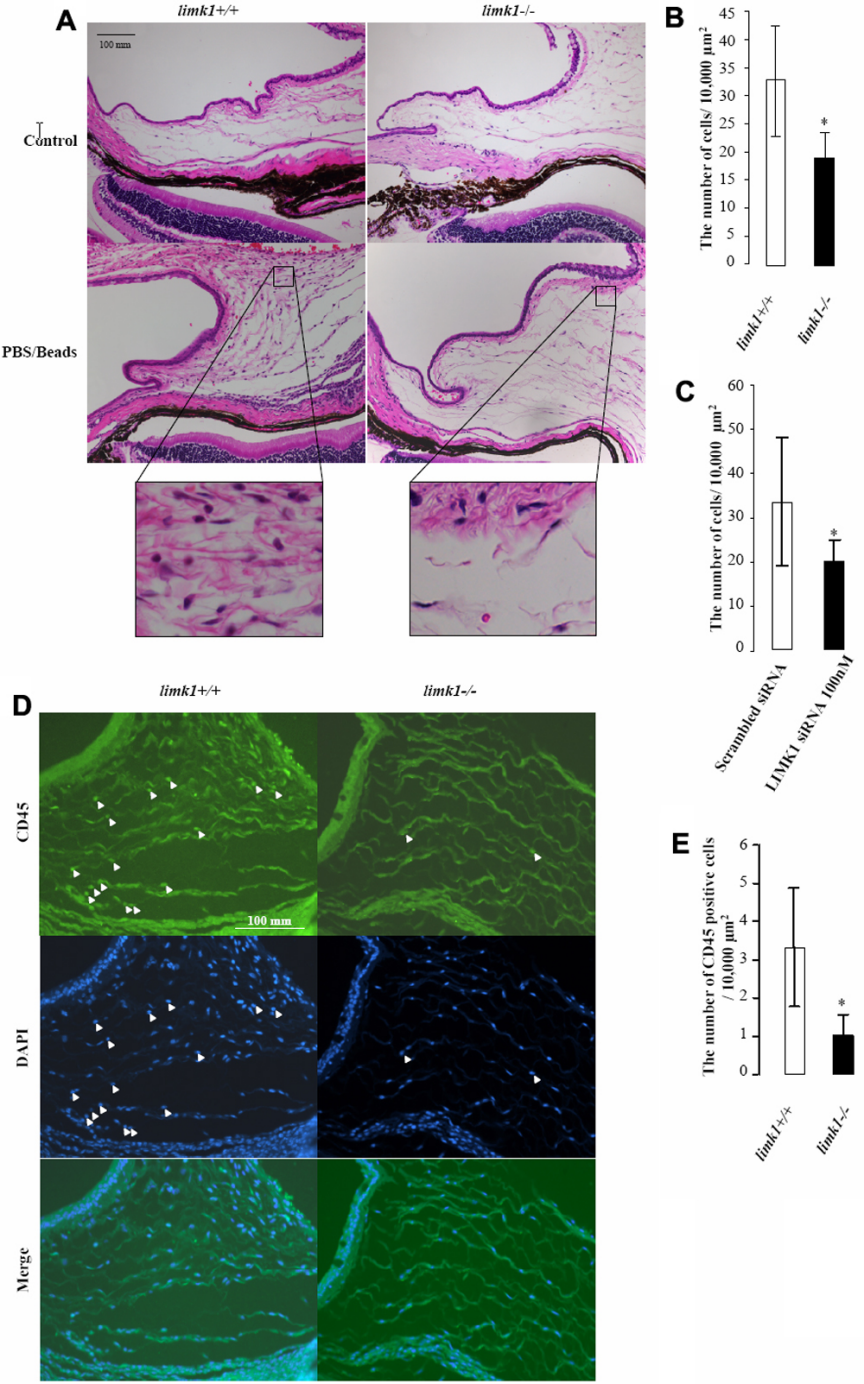
Suppression of inflammatory response by downregulation of *LIMK1* in a mouse model of ocular inflammation. **A**: Tissue sections from the mouse eyes of *limk1^+/+^* and *limk1^−/−^* genotypes treated with PBS/beads where indicated. **B**: Bar graph showing the number of inflammatory cells per 10,000 μm^2^ of subconjunctival area in PBS/beads-injected eyes of *limk1^+/+^* and *limk1^−/−^* mice. The asterisk indicates a p=0. 028 (n=4) compared with *limk1^+/+^* (n=5) specimens. **C**: Bar graph showing the number of inflammatory cells per 10,000 µm^2^ of subconjunctival area in eyes of mice treated with scrambled siRNA or *LIMK1*-targeted siRNA where indicated. The asterisk indicates a p=0. 024 (n=8) compared with scrambled siRNA controls. **D**: Immunofluorescence analysis of leukocyte infiltration in PBS/beads-injected *limk1^+/+^* and *limk1^−/−^* mouse eyes. Sections were stained with anti-CD45 antibody, and the cellularity was assessed by the co-staining with DAPI. White arrowheads indicate CD45-positive cells. **E**: Bar graph showing the number of leukocytes per 10,000 μm^2^ of subconjunctival area in the PBS/beads-treated eyes of *limk1^+/+^* (n=5) and *limk1^−/−^* (n=4) mice. The asterisk indicates a p=0.027. Experiments were performed 3 times.

Numerous round and large inflammatory cells were observed as anticipated underneath the conjunctival epithelium in *limk1^+/+^* 2 days after PBS/beads injection (Figure 4A,B). Quantitative analyses indicated that the number of inflammatory cells was markedly lower in *limk 1^−/−^* mice (Figure 4A,B).

To test whether direct application of *LIMK1*-targeted siRNA in the eyes would have the same anti-inflammatory effects, we injected anti-mouse *LIMK1* or negative control siRNA together with PBS/beads into the subconjunctival space in a masked manner. The robust inflammatory response in the mice injected with scrambled siRNA was similar to that with PBS/beads only (Figure 4B,C). By contrast, mice treated with *LIMK1*-targeted siRNA exhibited reduced inflammatory response as compared to the mice injected with scrambled siRNA (Figure 4C).

To analyze the nature of inflammatory cells observed upon injection with PBS/beads, tissue sections from *limk1^+/+^* and *limk1^−/−^* mice were stained with an antibody for CD45, a leukocyte marker. Consistent with that presented in Figure 4B, the data showed that the number of leukocytes upon the injection of PBS/beads in *limk1^−/−^* mice was significantly fewer than in the *limk1^+/+^* genotype (Figure 4D,E).

## Discussion

The present study demonstrates that siRNA specific to human *LIMK1* downregulated the protein expression in cultured human corneal fibroblasts. *LIMK1*-targeted siRNA application decreased the fibronectin deposition, reduced actin polymerization and focal adhesion formation, and retarded migration of the cells. Corneal fibroblasts constitutively express TGF-β, which is known to induce the expression of matrix molecules such as fibronectin and collagen type I [28,29] and facilitate migration of corneal fibroblasts [29,30]. Both of these are crucial steps in the wound repair process [31]. Therefore, our data suggests that *LIMK1* might be involved in the wound repair, regulating both transcriptional and migratory signaling pathways associated with TGF-β.

Several reports demonstrated the interaction between LIMK and TGF-β superfamilies. A direct association between LIMK1 and BMPR-II, a member of the TGF-β superfamily, was documented to result in influences on actin cytoskeleton dynamics [14,15]. Also, LIMK2, a member of the LIMK family, can be indirectly activated by TGF-β type I receptor through Rho and ROCK1 [16] to regulate actin assembly. In this report, we demonstrated another possible cross-talk between LIMK and TGF-β, notably in the wound repair signaling.

We observed that LIMK1 may be involved in the regulation of both migratory and transcriptional signaling pathways mediated by TGF-β. The influence of *LIMK1* downregulation on TGF-β-dependent cell migration may be explained by the impairment of effective actin cytoskeleton dynamics since *LIMK1* downregulation resulted in reduced actin polymerization, fibronectin deposition, and focal adhesion formation (Figure 3 and [1]). The links between LIMK1 and transcriptional pathways regulated by TGF-β however are less clear. The reduced production of fibronectin could be a result of diminished actin polymerization as it is known that ECM signaling is largely connected to actin cytoskeleton dynamics [32]. Alternatively, LIMK1 could also act directly on transcriptional pathways given the facts that LIMK1 possesses two LIM domains [33], which may bind DNA; and that there have been reports describing LIMK1 nuclear localization [34] and its direct effects on the protein expression [35].

We demonstrate that downregulation of *LIMK1* via genetic deletion or direct application of mouse *LIMK1*-targeted siRNA in the eyes of a mouse model significantly decreased ocular inflammatory response. As previously described [4], we injected PBS mixed with latex beads into mouse eyes to induce inflammation [8]. We observed in 2 days that both *limk1^−/−^* mice and wild type mice treated with *LIMK1*-targeted siRNA had much lessened inflammatory response, as evidenced by decreased infiltration of inflammatory cells such as leukocytes. The fibrosis response presumably was also reduced as it is related to the reduced inflammation (unpublished data), impeded fibroblast migration, and declined matrix deposition.

The siRNA injected into mouse eyes most likely targeted fibroblasts in the subconjunctival region. The precise mechanism of how siRNA was entering the cells is not known. The siRNA treatment reduced the inflammation, possibly in association with the attenuation of chemotaxis. As is shown in Figure 2 and Figure 3, *LIMK1* siRNA treatment retarded the migration and altered actin dynamics of corneal fibroblasts in culture. Downregulation of *LIMK1* in leukocytes was also observed to decrease leukocyte chemotaxis in Transwell assay, presumably via impairment of actin polymerization (unpublished data).

It is noteworthy that while adenovirus and other vectors may be employed for long-term inhibition of inflammation, transient knockdown by synthetic siRNA allows a better dosage control to minimize potential side effects. In our model a one-time administration of siRNA at a very early phase was sufficient to reduce the pathway of inflammation.

Ocular fibrotic wound response is a major cause of vision handicap and blindness, especially following surgical treatment for glaucoma [17,18]. Excessive post-operative scarring often leads to failure of filtration surgery. While the use of antimetabolites such as mitomycin C and 5-fluorourcil as conjunctival anti-fibrosis or anti-scarring treatments benefits some patients, these agents are associated with potentially blinding complications including hypotony, maculopathy, and infection [19-22]. Therefore downregulation of *LIMK1* might be an effective strategy in suppression and prevention of ocular inflammation and fibrosis.

To summarize, we report that *LIMK1*-targeted siRNA application in vitro resulted in decreased fibrosis, reduced actin polymerization, and retarded migration of fibroblasts, disclosing a possibility that LIMK1 may mediate TGF-β-dependent signaling during ocular inflammation. Also, downregulation of *LIMK1* via genetic deletion or direct application of *LIMK1*-targeted siRNA in the eyes of a mouse model markedly decreased ocular inflammatory response. The in vitro and in vivo results underscore the potential of a novel therapy for preventing the inflammatory response and fibrosis or scarring in ocular diseases and after glaucoma filtration surgery. This approach may also have applications for other surface tissues, including the skin.
